# Geographical Origin Assessment of Extra Virgin Olive Oil via NMR and MS Combined with Chemometrics as Analytical Approaches

**DOI:** 10.3390/foods11010113

**Published:** 2022-01-01

**Authors:** Francesca Calò, Chiara Roberta Girelli, Selina C. Wang, Francesco Paolo Fanizzi

**Affiliations:** 1 Department of Biological and Environmental Sciences and Technologies, University of Salento, Strada Provinciale Lecce Monteroni, 73100 Lecce, Italy; francesca.calo@unisalento.it (F.C.); chiara.girelli@unisalento.it (C.R.G.); 2 Department of Food Science and Technology, University of California Davis, One Shields Avenue, Davis, CA 95616, USA; scwang@ucdavis.edu

**Keywords:** extra virgin olive oil, geographical origin, metabolomics, nuclear magnetic resonance (NMR) spectroscopy, mass spectrometry, molecular fingerprinting, isotope ratio, elemental profiling, chemometrics

## Abstract

Geographical origin assessment of extra virgin olive oil (EVOO) is recognised worldwide as raising consumers’ awareness of product authenticity and the need to protect top-quality products. The need for geographical origin assessment is also related to mandatory legislation and/or the obligations of true labelling in some countries. Nevertheless, official methods for such specific authentication of EVOOs are still missing. Among the analytical techniques useful for certification of geographical origin, nuclear magnetic resonance (NMR) and mass spectroscopy (MS), combined with chemometrics, have been widely used. This review considers published works describing the use of these analytical methods, supported by statistical protocols such as multivariate analysis (MVA), for EVOO origin assessment. The research has shown that some specific countries, generally corresponding to the main worldwide producers, are more interested than others in origin assessment and certification. Some specific producers such as Italian EVOO producers may have been focused on this area because of consumers’ interest and/or intrinsic economical value, as testified also by the national concern on the topic. Both NMR- and MS-based approaches represent a mature field where a general validation method for EVOOs geographic origin assessment could be established as a reference recognised procedure.

## 1. Introduction

Extra virgin olive oil (EVOO) is a high-value product due to its excellent nutritional properties and organoleptic characteristics, which are appreciated for their positive effect on human health. It is an important part of the Mediterranean diet due to the beneficial effects related to fat composition [1,2,3,4], which includes monounsaturated, polyunsaturated, and saturated fatty acids, mainly in the form of esters with glycerol (triglycerides), which represent more than 98% of the total olive oil content [5]. Moreover, EVOO is also a good source of antioxidants such as polyphenols and tocopherols [1], representing the minor components, together with sterols, volatile compounds, terpenols, acylglycerols and other hydrocarbons [6]. All of these elements have protective effects on our well-being and also help to prevent diseases such as cancers, diabetes, and autoimmune and cardiovascular illness [3]. Olive oil is a complex multi-component food matrix whose analysis is not a simple task; its characterization is made even more difficult by the increasingly widespread problem of adulteration with low-quality products [7] and in some cases also with the addition of other low-cost edible vegetable oils of uncertain origin [8]. Although the oil may be of similar quality, specific EVOOs from certain countries are valued more than those originating in other countries [9]. Therefore, characterization and certification of product origin are of importance, especially with the continual increase in the number of EVOO-producing countries and the attention placed on this product worldwide. Presently, this specific consideration focuses not only on food safety and quality control but also on the declared geographical origin authenticity assessment [10]. It should also be kept in mind that the food traceability may also have an impact on health, as well as on customer confidence on specific suppliers and product quality control, including the geographical origin assessment. Certainly, a case of fraud can have serious consequences with significant impact on individual customers as well as on the entire market [11]. Therefore, the constant regulation amendment related to EVOO characteristics aims at facilitating product marketing, promoting truth labelling and establishing general rules for correct claims [12]. The reasons for assessment of the geographical origin for EVOOs could be considered as essentially related to the mandatory legislation and/or the obligation of true labelling. In fact, the label should provide consumers with the necessary product information to understand the EVOO’s characteristics including its geographical origin.

The European Commission (EC) first established (Reg. N. 1019/2002) the indication of the geographical origin as an optional label [13]. Subsequently (Reg. N. 182/2009), in order to guarantee full product traceability and complete consumer protection, EC introduced the compulsory labelling of EVOOs with the country of origin indication (COO) [14], in accordance with the Italian policy (2007) that for some time made this information mandatory at national level [15,16]. It should also be considered that the European Union (EU), through the Regulations 2081/92 [17] and then 510/2006 [18], had already introduced provisions on the protection of geographical indications and origin designations for agricultural and food products [19,20]. Since March 2009, the European Union Regulation declared that labelling of EVOOs was compulsory in all European countries with a clear indication of the geographical origins of the olives used for the production [14]. More recently, the EC Regulation 1151/2012 implementing rules on marketing standards for olive oil determined the mandatory nature of origin labelling [21] This decision highlighted that the characteristics of the product are also related to the geographical origin and the experience gained by operators and administrations involved in the production. Thus, the mandatory labelling, which must give precise information about olive oil’s geographical origin is required, although an official validated methodology for specific origin assessment is still missing [22,23]. The purpose of such mandatory labelling is to protect, in the food sector, the position of the European consumers, also guaranteeing the principle of loyalty, in the market competition, with the complete traceability of the product [24]. Despite mandatory geographical origin labelling of EVOOs in Europe, there are different indications for this issue in countries outside the European Community. In the United States (US) there is no obligation, but if the geographical origin of the product is reported, this should be also verifiable ensuring label reliability. On the other hand, if the geographical indication is considered a generic name in the US, it therefore cannot be further otherwise protected [25]. Currently, a new legislation (Assembly Bill 535) [26] is being introduced in California, in which almost all US olive oil is produced, aiming to add restrictions on using the word “California” on olive oil labels. There are existing California laws that prohibit the term “California olive oil” on labels in which the oil is not produced from 100% California-grown olives; however, it the new bill is enacted, it would prohibit the use of “California olives”, “California olive oil”, or other similar terms in brand names, products, or any material associated with products that are not produced from 100% California-grown olives. Additionally, if passed, the bill would apply similar restrictions for olive oil produced in certain Californian regions unless 85% olive oil had been produced in the named region. In the US, the labelling of extra virgin olive oil is regulated by the Code Federal Regulations, Title 21 “Food and drugs” [27], according to which the label must include information that shows the countries of origin as “Made in” or “Product of” if the product is a blend of oils from different geographic origins. Recent solutions proposed by the American Olive Oil Producers Association (AOOPA) and the North American Olive Oil Association (NAOOA) were shown during the 2021 AOCS Olive Oil Expert Panel Meeting [28]. According to NAOOA, the geographical origin on the label should be regulated as proposed by AOOPA, so it must be truthful, accurate, and not false or misleading in any way, but with more details on Country, State, and Estate requirements. In Asian countries such as Japan, according to the JAS (Japan Industrial Standards) law [29], information such as the country of origin should be reported in the label. The country is the one where the substantial processing is carried out, but specific guidelines allowing key processing identification are still missing. In fact, for EVOOs, some Japanese importers prefer to indicate as country of origin the location of blending and bottling rather than that of pressing. For this reason, in the Japanese market, there are “Made in Italy” olive oils also containing products from other countries and bottled in Italy. Mandatory labelling for EVOO is also required in Arab countries [30], but information such as geographical origin remains optional. With multiple sources of standardization and different interests worldwide, it is difficult to find harmonization in the indication of geographical origin, specifically for food products.

**Figure 1:**
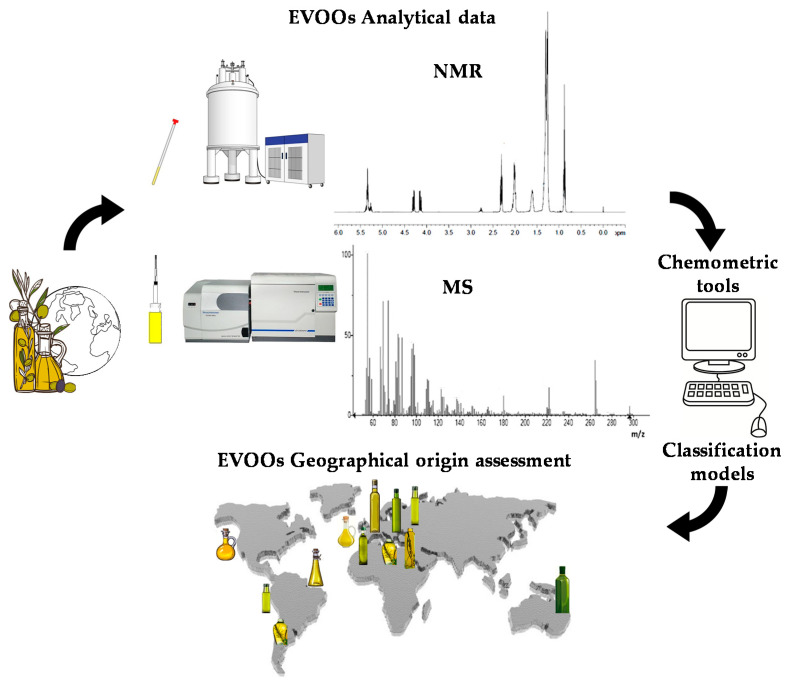
Comprehensive mechanism-based scheme summarizing the application of NMR and MS techniques associated with chemometric tools in the specific subject of EVOOs’ geographical origin analysis.

Due to the economic and political interests of the various countries involved in the EVOO market, there is a growing interest in the development of a technique able to give more detail about the origin of this product [31,32]. Scientific research has been done to identify analytical techniques to detect food fraud and guarantee the authentication of EVOO and the presence on the market of products characterized by labels with truthful information. Even though there are different studies on the subject, a method for certifying the EVOOs’ geographical origin has not yet been established in the science literature. In recent years, increasing attention has been given to several analytical techniques capable of assessing the characteristics of EVOO through the study of its chemical-physical and organoleptic properties. Genetic approaches have also been used, although these latter allow investigation of the varietal rather than geographical origin of the product [33,34]. Besides the cultivar contribution, the effect of pedoclimatic conditions and agricultural practices is much better analysed by looking at the metabolic profiles of the product [35]. Indeed, olive trees of the same cultivar can be planted in several countries and, despite being characterized by the same genetics, the oil produced will be different. For this reason, notwithstanding the well-established importance of genetic characterization, assessment of the geographical origin of EVOO is preferably studied using analytical techniques dedicated to metabolic rather than genomic profiling. Currently, EVOO metabolic profiling generally takes advantage of two analytical techniques, which have been considered in the present review: nuclear magnetic resonance (NMR) [35] and mass spectrometry (MS) [36,37,38]. These techniques are usually associated with chemometrics methods involving metabolomics with the application of statistical analysis to spectroscopic chemical data [39,40,41]. A comprehensive mechanism-based scheme summarizing the application of these techniques in the specific subject of EVOOs’ geographical origin analysis is depicted in Figure 1. The characteristics of the two analytical techniques have already been fully described in the literature [42,43]. A specific description of advantages and shortcomings for NMR and MS techniques is summarized in Table 1. The results obtained in EVOO geographical origin assessment, including the building and use of specific databases dedicated to this purpose, are reviewed in the present work.

## 2. Nuclear Magnetic Resonance (NMR)-Based Studies

The nuclear magnetic resonance (NMR)-based metabolomic approach represents a powerful tool for assessment of EVOOs’ origin and authenticity [44]. NMR is often used to analyse foodstuff, including olive oil, providing a complete metabolic profile with qualitative and quantitative information on its major and minor components [45,46]. NMR spectroscopy can be thought of as a very powerful camera that is able to take a snapshot of all the molecular components present in a specific complex matrix. The application of chemometric methods to classify the analysed samples, using NMR data, allows the natural variability of the chemical composition of complex matrices to be take into account, including those related to the geographical origins in the case of EVOOs [20]. The NMR spectroscopy associated with MVA, allows EVOO’s metabolic profiles to be to defined and clustered, accounting for different parameters such as cultivars, pedoclimatic condition, temperature and humidity, growing areas, and agriculture practices [1,35]. Specific clustering, usually observed according to EVOO cultivars and/or geographical origin, can be also used for prediction purposes and traceability assessment. This technique requires an easy sample preparation (without the need for preliminary separations), quickly providing a complete metabolic profile of the analysed matrix, including olive oil [47]. The great potential of this spectroscopic analysis lies not only in its non-destructive nature but also in the fact that NMR ensures a univocal correspondence between specific signals of the metabolites and the metabolites themselves, resulting specific product fingerprinting with structural information on the metabolites. In addition to high precision and a remarkable degree of reproducibility, NMR profiling provides a vast number of data in a single analysis [48]. This analytical technique, associated with MVA, also allows tailor-made databases to be obtained [7] for EVOO sample discrimination according to cultivar and/or geographical origin [49,50]. A range of specific NMR signals, representative of selected metabolites, often allows good sample classes discrimination. The main drawback of NMR spectroscopy is its intrinsically low sensitivity, as well as purchase and maintenance costs due to the use of cryomagnets [42]. On the other hand, it should be considered that modern NMR spectrometers take advantage of high resolution instruments [50] with acquisition of multiple scans, operating at high magnetic fields, using cryoprobes [51] and sometimes hyperpolarization methods [42]. Very meaningful NMR data need to be acquired, possibly at high fields [52]; an increase in the magnetic field intensity results in higher spectra resolution and easier metabolite identification. A further promising application area could be also related to the very recent use of a low field NMR instrument, usually based on permanent magnets [53,54,55]. This latter takes advantage of both modern Fourier transform (FT)-based acquisition techniques as well as low operational costs [56].

**Figure 2:**
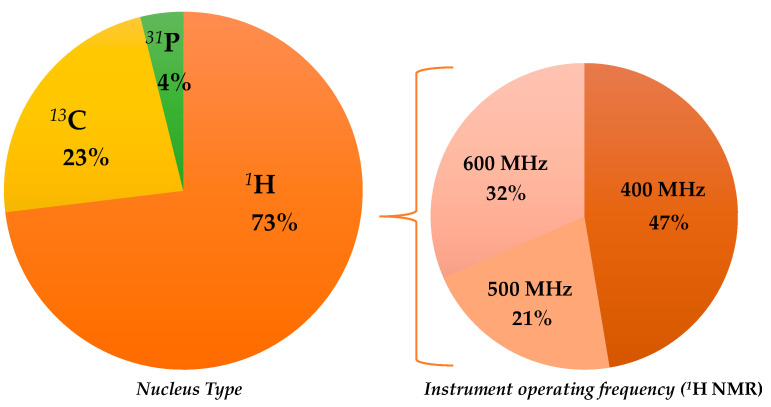
Graphical representation of the partition of selected nuclear magnetic resonance (NMR)-based studies on extra virgin olive oils (EVOOs)’ geographical origin assessment.

There are several interesting published works describing NMR-based statistical protocols as a scientific tool in EVOO geographical origin assessment; some of them have been already included in previous general reviews on olive oil analyses [35,38,45,57]. In the present review, only the selected papers related to the specific topic of EVOO geographical origin assessment will be described. These are reported, with the summarized major outcomes (classification model realization; prediction test execution; molecular markers identification) in chronological order in Table 2, and will be discussed accordingly in the following paragraphs, considering the observed nuclei and, in the case of ^1^H, the NMR instruments used (Figure 2). The specific molecular markers identified in the listed NMR studies are summarized in Table 3.

### 2.1. ^1^H NMR Spectroscopy

Although the first NMR paper, which was related to EVOO geographical origin assessment, used ^13^C spectroscopy (see below), ^1^H NMR was shown to be the one used most. The first scientific publication related to the use of the ^1^H NMR technique for the classification of EVOOs according to their geographical origin dates back to the year 1998 and used a 600 MHz instrument. Thereafter, lower field instruments were also used (500, 400 MHz) with 400 MHz being shown to be the most popular in the recent years. Therefore, this review will discuss ^1^H spectroscopy-based literature, according to the used instrument, in the following order: 600, 500, and 400 MHz.

#### 2.1.1. 600 MHz ^1^H NMR

Sacchi et al., 1998 [59], published a research study on the characterization of Italian EVOOs, first using ^1^H NMR spectroscopy and MVA, also explaining the potential strong contribution of this technique to the authentication of the geographical origin. Then, PDO products were reported in the early studies of Mannina et al., 2001 [63], in which ^1^H NMR spectroscopy was used for the geographical characterization of Italian EVOOs. Mannina et al., 2005 [68], also used ^1^H NMR spectroscopy to investigate PDO products originating from a northern Italian region (Veneto, North East Italy). Thereafter, the study conducted by Schievano et al., 2006 [70], indicated the possibility of discriminating by ^1^H NMR, even EVOOs from different micro-areas (Veneto and Lombardia banks of Garda lake, Italy) within the same PDO zone (Garda). Then, Mannina et al., 2010 [73] used ^1^H NMR spectroscopy to analyse EVOOs coming from several Mediterranean areas (Italy, Spain, France, Greece, Cyprus, and Turkey). In this case, the NMR data associated with MVA allowed researchers to discriminate between Ligurian (North West Italy) and non-Ligurian olive oils. Longobardi et al., 2012 [76], used ^1^H NMR fingerprinting combined with MVA for the classification of EVOOs from three different regions of Apulia in Italy (Dauno, Terra di Bari, and Terra d’Otranto) and four different regions in Greece (islands of Kefalonia, Kerkira, Lefkada, and Zakinthos). Interestingly, this appears to be the first paper where multisuppressed ^1^H NMR experiments, able to enhance the minor components with respect to major components in the acquired spectra, were reported in the geographical origin EVOOs characterization. Aghemo et al., 2012 [78], using ^1^H NMR along with GC, characterised the fatty acid profile of Piedmont EVOOs for the first time and compared them to other oils from five Italian regions. A good separation between EVOOs produced in the North of Italy from those of Central and Southern regions resulted from this geographical investigation. A specific association of ^1^H NMR analysis of Italian PDO EVOOs combined with the study of the isotopic composition was reported in the work of Camin et al., 2016 [85]. The additional use of NMR data allowed, using multivariate statistical analysis, highly correct discrimination (98.5%) of olive oils from Italy and Tunisia. Chemometric models based on NMR data were also used for discrimination of the EU Protected Designation of Origin Sardinian oils by Culeddu et al., 2017 [90]. The obtained results constituted the first step towards a thorough characterization of several monovarietal Sardinian oils. Özdemir et al., 2018 [92], carried out the authentication of Turkish and Slovenian olive oils on the basis of ^1^H NMR profiles. It was found that known and local cultivars harvested in different geographical locations were discriminated mainly based on their composition of phenolic compounds, terpenes, and diacylglycerols. Indeed, the EVOOs’ phenolic profile, obtained by LC-MS, provided a fingerprint capable of distinguishing EVOOs’ geographical origin and authenticity, as mentioned by Olmo-García et al. [100]. A recent characterization and discrimination of Italian EVOOs according to the geographical areas of the North, the Islands, and the Centre-South was performed by Ingallina et al., 2019 [22], using a combination of ^1^H NMR spectroscopy and chemometric analysis in a single classification model. The use of the NMR technique associated with chemometric analysis as an appropriate analytical approach to guarantee the traceability and authenticity of the EVOO was suggested to the regulatory authorities by the review of Consonni and Cagliani, 2019 [45]. Özdemir and Bekiroğlu 2019 [94] explained the great potential of ^1^H NMR spectroscopy, coupled with multivariate statistical analysis, also to discriminate Turkish Gemlik olives cultivated in a PDO region from those cultivated in non-PDO regions.

#### 2.1.2. 500 MHz ^1^H NMR

Five different production countries of EVOOs (Greece, Italy, Spain, Tunisia, Turkey) were considered by Rezzi et al., 2005 [69] for origin assessment using ^1^H NMR observed on a 500 MHz instrument. This appears to be the first paper in which NMR methods were used for characterization of the geographical origin of EVOOs that also included other major producers besides Italy. Papadia et al., 2011 [74], also used ^1^H NMR spectra to characterize EVOOs from five different Apulian areas, focusing on possible correlations with growth soil analyses. Del Coco et al., 2012 [77], compared Italian products commercially available in the US with Apulia Italian EVOOs as well as Spanish, Greek, and Tunisian ones, revealing the possible discrimination among samples. Investigations by ^1^H NMR on EVOOs obtained from secular olive trees, in the Apulia Italian region, were also reported by Del Coco et al., 2013 [79]. Differences in chemical composition and NMR profiles of EVOOs were related not only to cultivars but also to geographic areas and seemed to justify a larger biodiversity maintenance of Apulia secular germplasm. The results of Rongai et al., 2017 [46], also suggested the use of the ^1^H NMR-based metabolic profile, combined with multivariate analysis, for the evaluation of the possible correlation of EVOO characteristics with climatic data, as well as the prediction of their geographical origin. Marked differences between Italian and other foreign countries EVOO samples were observed, in particular when the three studied Italian regions (Tuscany, Sicily, and Puglia) were considered separately. Higher differences in mean rainfall and temperature were generally associated with a more constant discriminating capacity of the statistical models studied.

Interestingly, the use of lower field instruments first appeared in scientific works where both 500 and 400 MHz were used. Sacco et al., 2000 [61], first reported their studies on Italian EVOOs from different areas of the Apulia region (Southeast Italy), the major producer in the country, demonstrating that ^1^H NMR data of phenolic extracts (obtained by both 500 and 400 MHz instruments) allowed a classification according to the geographical origin of the samples. Ok, 2013 [80], published a study reporting a quantitative ^1^H NMR (500 and 400 MHz) analysis used also for discriminating olive oil samples from Turkey, Jordan, Palestine, and Libya. Statistical methods allowed discrimination based on territorial origin, highlighting that this screening possibility did not require additional specific olive oil analyses. Interestingly, in the same study, two-dimensional (2D) NMR ^1^H DOSY experiments were also used and proposed as a tool for origin assessment based on minor constituents.

#### 2.1.3. 400 MHz ^1^H NMR

Del Coco et al., 2014 [81], used ^1^H NMR to characterize EVOOs from a subarea (Salento) of the Apulia region using only a 400 MHz instrument. The age of the trees was also investigated as a feature related to the oil metabolic profile. Higher polyphenols and polyunsaturated fatty acid contents were found in EVOOs originating from young compared to secular trees. ^1^H NMR Spectroscopy and MVA of monovarietal EVOOs were also used by Del Coco et al., 2014 [82], to evaluate the modulation of Coratina-based blends, providing the opportunity to address tastes for blended EVOOs by using oils from a specific region or country of origin. ^1^H NMR spectroscopy and chemometrics were also used by Del Coco et al., 2015 [83], to investigate potential differences of South Apulia (Salento) Italian EVOOs related to the major and minor chemical composition. Once again, the results showed the influence of pedoclimatic differences on EVOOs originating from specific micro-areas. The denomination of protected origin (PDO) “Terra di Bari” from Apulia (Italy) was studied by Del Coco et al., 2016 [86]. Statistical analyses based on ^1^HNMR data demonstrated possible commercial PDO origin assessment and the ability of the models to discriminate, according to micro-area, pedoclimatic effects, as well as small geographical zones within the same PDO area. Girelli et al., 2016 [87], recently introduced the use of reference monocultivar oils from selected Italian Regions (Apulia and Calabria) for the assessment (by a ^1^H NMR-MVA based model) of specific Italian EVOOs blends containing the same cultivars from specific geographical regions. This methodology offered a simple and clear tool to buttress the labelled EVOOs geographical origin, also for commercial purpose, despite the lack of an official procedure supporting the compulsory origin declaration stated at the EU level. In a further research work of Girelli et al., 2016 [50], the ^1^H NMR-based metabolomic approach was used to evaluate EVOOs originating from the provinces of Bari and Foggia (Apulia region, Italy) during two consecutive harvesting seasons. The influence of the harvesting season on the oil metabolic profiles for the different cultivars in the specific geographical areas were analysed to assess the stability of monocultivar-based discrimination models, which were shown to be reliable especially for Coratina-based blends. These models, obtained using monocultivar oils from specific geographical areas, were also tested for their robustness with respect to the variations in the instrument field used or the use of NMR rather than other data describing EVOOs (NIR, classical analyses). Piccinonna et al., 2016 [88], demonstrated the comparable results obtained using a 400 MHz compared to a 500 MHz spectrometer and even a possible merging of both NMR data to obtain a single model. On the other hand, Binetti et al., 2017 [89], reported the superior performance of models based on NMR with respect to NIR and merceological data as assessed by artificial neural networks. Girelli et al., 2017 [91], conducted a study on Tunisian and Italian (Coratina) EVOO blends using ^1^H NMR associated with MVA to investigate the possible Tunisian EVOO traceability in the EEC market blends. A series of binary mixture blend oils were obtained, starting from specific batches of Italian (Coratina) and Tunisian (Chemlali, Chetoui) oils. The models built showed the linear relationship between the ^1^H NMR-based blend profiles and the percentage compositions. In the case of Tuscany PGI, a specific work by Girelli et al., 2018 [49], demonstrated the ability of ^1^H NMR-based statistical models to discriminate, for the same cultivars, also different geographical areas within the same PGI region. This work concerns oils obtained from cultivars and specific geographical areas for the production of EVOO PGI from Tuscany in Italy, a region characterized by high pedoclimatic variability. The analysis of ^1^H NMR profiles with MVA, based on minor components, described by Winkelmann and Küchler, 2019 [93], also proved very useful for the reliable classification of EVOOs from Italy, Greece, and Spain, as the main producing countries in the Mediterranean area. The obtained statistical model allowed classification for oils from the three considered origins, and the analytical approach was also suggested for routine evaluations. Girelli et al., 2020 [23], with the same approach of a previous study [87] based on ^1^H NMR data, reported the implementation and use of a specific Italian monocultivars database from selected geographic origins over four harvesting years. The obtained models were used to classify commercial 100% Italian, Coratina-based, blended EVOO samples. Minor component contribution (combined zg-noesy spectra) was taken into account, in additional to the major lipid fraction (standard zg spectra) allowing for the performance evaluation of different MVA models. Possible correlation of blend EVOOs classification in the models with specific components content and organoleptic characteristics (bitterness, pungency, fruitiness) was also reported. The Lukic et al., 2020, study [98] has recently shown that the NMR technique, together with liquid chromatography/mass spectrometry (LC-ESI-MS/MS, LC-ESI-IT-MS), can also support market claims relating to the geographical origin of the product, as well as testify quality and justify price. The relationship between lipid composition and geographical origin was evaluated for two classes of EVOOs according to the origin of purchase: monocultivar DOP Italian EVOO from family farms and commercially blended EVOO from supermarkets. The results of this study proved the heterogeneity of the studied oils sold in relation to their different origins. Calò et al., 2021 [99], studied commercial international EVOOs blends, originating from different countries (Italy, Tunisia, Portugal, Spain, and Greece), using standard and multi-suppressed ^1^H-NMR experiments. The possible correlation of the EVOOs blends’ characteristics (as defined by their ^1^H-NMR profiles) with the blend composition was investigated. International blends’ features strongly correlated with the content of Italian EVOO constituents, suggesting the possible heavy influence of Coratina-based oils, for the studied dataset.

### 2.2. ^13^C NMR Spectroscopy

In 1997, the first ever scientific publication related to the use of the NMR technique for the geographical origin assessment of EVOOs concerned the ^13^C nucleus type. Shaw et al., 1997 [58], focused on the discrimination of EVOOs’ origin using ^13^C NMR spectroscopy, associated with MVA, with the aim of avoiding product adulteration with cheaper oils and untrue label declarations. The possible discrimination of Italian olive oils by geographical origin, as well as by cultivars, was then investigated by Vlahov et al., 1999 [60], using distortionless enhancement by polarization transfer (DEPT) pulse sequence to set up a quantitative high-resolution ^13^C NMR method. Scientific works related to the NMR analysis of Italian PDO olive oils were published starting in 2001. Vlahov et al., 2001 [62], used ^13^C spectra to discriminate olive oils from different Italian geographical areas of production, including PDO areas. Many papers, selectively focused on Italian EVOOs, further appeared in 2003 originating from the regions of Sicily [65] and Apulia [67]. Some of them were related to PDO products, analysed by ^13^C NMR, from the Apulia region [66]. Rongai et al., 2019 [95], selectively applied ^13^C NMR analysis for the geographical characterization and possible discrimination of EVOOs produced in some Italian regions (Abruzzo, Calabria, Lazio, Liguria, Puglia, Sardinia, Sicily, and Tuscany) and reported correlations of the observed differences with different climate conditions.

#### ^13^C Together with ^1^H NMR Spectroscopy

Interestingly, ^13^C data together with ^1^H ones were first considered by Mannina et al., 2001 [64], for geographical origin assessment of olive oils from areas other than Italy (such as Argentina). D’Imperio et al., 2007 [71], were able to distinguish northern, central, and southern EVOOs of another Italian PDO area (Lazio, central Italy), using ^1^H and ^13^C NMR data, coupled with MVA. Then, high-resolution magic angle spinning (HR-MAS) was first introduced by Corsaro et al., 2015 [84], using one- and two-dimensional NMR experiments for Mediterranean diet foods analyses. These included PDO EVOOs from Sicily (Italy), and the approach used allowed researchers to identify and quantify the main metabolites possibly related to the geographical origin. Vicario et al., 2020 [96], characterized EVOO samples from a specific Italian area (Tuscany) combining ^1^H and ^13^C NMR with near UV-Vis absorption spectroscopy. The identified and quantified different chemical components, related to EVOOs’ nutritional and quality properties, were correlated with specific features of the cultivation area. The Arslan and Ok, 2019, review paper [57], starting from previous works [80,92] from the same group, focused on the screening of Turkish olive oils according to their chemical content and possible comparisons with oils with other nations of origin (Spain, Italy, Greece, and Tunisia). This work highlighted the importance of NMR when dealing with oil adulteration issues or specific characterizations (olive cultivars, geographical locations, harvest season, and soil quality). The discrimination of Maltese from non-Maltese EVOOs was successfully carried out by Lia et al., 2020 [97], using statistical models based on ^13^C, simple ^1^H zg30, and multi suppressed ^1^H NOESY NMR spectra.

### 2.3. ^31^P NMR Spectroscopy

Fewer scientific works exist related to the use of ^31^P NMR spectroscopy generally, together with ^1^H NMR technique. In 2008, focusing on selected characterization according to geographical micro-areas, EVOOs from different Greek regions were studied using ^1^H and, for the first time, ^31^P NMR spectroscopy [72]. Then, Agiomyrgianaki et al., 2012 [75], further reported a study on Greek EVOOs from different regions with ^1^H and ^31^P NMR spectroscopy. The influence of cultivars on the EVOO samples’ characteristics according to harvest year and geographical origin emerged from this work.

## 3. Mass Spectrometry (MS)-Based Studies

Mass spectrometry (MS) along with other analytical techniques and chemometric evaluations have been successfully employed for the quality control of oils and fats [101]. This analytical method is very often combined with other separation techniques to obtain a complete metabolomic profile suitable for MVA. The combination of MS with other techniques has proved very useful in the metabolic fingerprinting of various vegetable oils, including EVOO. Besides being a useful tool applicable to the quality assessment and detection of adulterations, this type of analysis is also a promising method for certifying geographical origin. Nevertheless, for the MS data, a correct correlation study of the molecular differences responsible for oils discrimination also requires a large database providing more complete information (such as changes in chemical composition due to the year of collection, environmental conditions, and methods of extraction). Many literature studies have focused their attention on the application of MS-based approaches used for geographical origin identification and discrimination of EVOO samples (Table 4). Some recent works also considered the use of the same techniques for geographical and/or botanical origin assessment of olive oils and virgin olive oils [102,103,104]. Specific compounds (such as triacylglycerol, volatile compounds, phenols) even present in low concentrations can be identified by MS. These are often considered as markers and can be used to characterize and differentiate olive oils based on geographic origin as well as olive cultivar. Indeed, the identification of the volatile composition of EVOO is mainly used as a mean of characterization and authentication, also taking advantage of headspace solid-phase micro extraction GC-MS (HS SPME-GC-MS) [105]. Moreover, the complete sterol and polyphenol profile by ultra-performance LC tandem MS method with electrospray ionisation (UHPLC-ES-MS/MS), coupled with MVA, appears to be a very promising tool for discriminating PDO EVOO samples according to their different geographic origins [106]. Considering these aspects, as demonstrated in several published scientific papers (Table 4), the authentication of EVOOs could be possible by monitoring specific markers, using cost-effective methods based on less sophisticated and inexpensive mass spectrometers such as ultra-performance LC with electrospray ionisation/quadrupole time-of-flight MS (UHPLC-ES/QTOF-MS) [107]. The major advantage of MS is the intrinsic high sensitivity of the analytical technique. Moreover, in combination with chromatography (such as liquid LC and gas-phase GC separation), it is a very selective and superior tool for targeted analysis. [42]. On the other hand, compared to NMR spectroscopy, MS data are less reproducible. Different ionization methods are required to maximize the number of detected metabolites. Gas chromatography usually requires sample derivatization, so it can be time-consuming and the sample cannot be recovered. However, it only requires a very small amount of sample. The intensity of the MS line is often not correlated with metabolite concentrations, as the ionization efficiency is also a determining factor [42].

**Figure 3:**
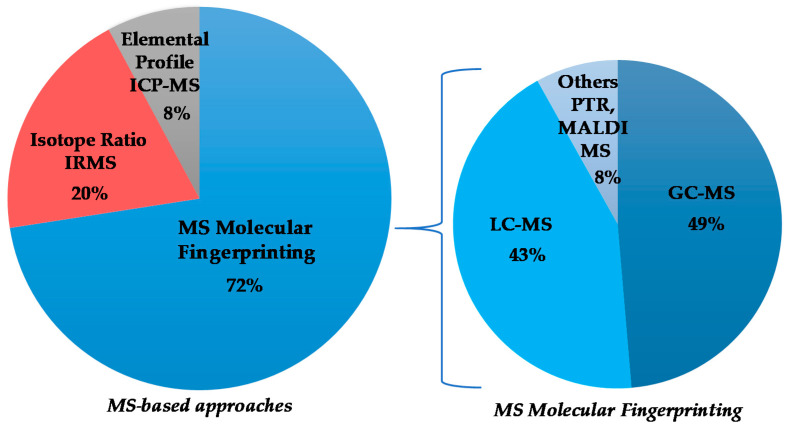
Graphical representation of the partition of selected mass spectrometry (MS)-based studies on extra virgin olive oils (EVOOs)’ geographical origin assessment.

Although the numerically most relevant applications of MS spectroscopy for the geographical origin EVOO assessment are related to molecular fingerprint, in the present review, MS-based isotopic ratios as well as elemental profiles determinations have been considered. The published works are chronologically listed in Table 4, with the major outcomes summarized (classification model realization; prediction test execution; molecular marker identification) and will be discussed accordingly in the following paragraphs, considering the different MS-based approaches and, in the case of MS molecular fingerprinting, according to the specific techniques (Figure 3). The specific molecular markers identified in the listed MS studies are summarized in Table 5.

### 3.1. MS Molecular Fingerprinting

#### 3.1.1. GC-MS

The first scientific paper appeared in this field of the molecular fingerprinting with MS verifying the declared EVOO geographical origin is by Salter et al., 1997 [108], which focused on the determination of Italian EVOOs’ geographical origin using pyrolysis-assisted MS analysis and artificial neural networks. This was the first report, entirely based on untargeted fingerprinting, where the MS data demonstrate the ability to discriminate Italian olive oils according to the region of origin using an artificial neural network. Then, almost a decade later, Cerrato Oliveros et al., 2005 [109], published the second paper on this topic. Interestingly, also in this case, the authors investigated the MS-based discrimination of different EVOOs’ geographical origin from five Mediterranean areas (Italy, Greek, Spain, and Tunisia) without performing any chromatographic separation. This was done by optimizing a new headspace MS instrument equipped with a sample introduction system directly coupled with a mass detector and analysing the obtained MS data with chemometric methods. Cavaliere et al., 2007 [110], demonstrated that chemical ionization MS with an ion trap (IT) provided an GC-MS apparatus associated with LDA and other statistical tools such as the Kruskal–Wallis and the Wald–Wolfowitz tests, allows the identification of specific markers for oils from different geographical areas. In this case, Italian EVOOs (Calabria region) could be discriminated from the Tunisian EVOOs using quantitative data. In the work of Lớpez-Feria et al., 2008 [112], the headspace MS analysis (HS-MS) was used for sensory characterization and classification of EVOOs and PDO according to olive variety and geographical origin (Spain and Italy). The use of HS-MS coupling was shown to be appropriate for routine control, reducing time for sample processing and analysis and giving excellent prediction results with chemometric models. Casale et al., 2010 [113], used the combined data from electronic nose, UV–visible and NIR spectroscopy to build a-class discriminating chemometric model for EVOOs from Liguria region (North-western Italy). The analysis of volatile compounds performed by Pouliarekou et al., 2011 [115], using headspace solid-phase microextraction GC MS (HS-SPME-GC MS) was found to be useful for characterizing and classifying EVOO samples from western Greece. In particular, using the MS data and a chemometric approach, the samples were satisfactorily classified based on their geographical origin (87.2%) or used cultivar (74%). From this study, it emerged that the typical environmental conditions specific to the considered geographical areas, as well as the harvesting period, clearly affect the EVOO samples’ characteristics and their possible discrimination. HS-SPME-GC MS analysis coupled with MVA was also used in the work of Pizarro et al., 2011 [116], allowing the identification of the most discriminating volatile marker compounds useful for the assessment of the geographical origin of EVOOs. In this case, a perfect discrimination was carried out for different Spanish oils from specific geographical regions (La Rioja, Andalusia, and Catalonia). Kandylis et al., 2011 [117], using the SPME GC MS technique, performed a study on the effects of the geographical origin, irrigation, and degree of ripeness of Koroneiki olives on the profiles of volatile compounds isolated from monovarietal oils from Crete and Tunisia. The tested samples showed different volatile profiles, allowing perfect discrimination between Cretan and Tunisian EVOOs. The results also indicated that, in the specific study, primary maturity and geographic origin rather than irrigation conditions affected the EVOOs’ volatile profile. The correlations between the sensory attributes, evaluated by a panel test, and the presence of specific volatile compounds, characterized by HS-SPME-GC-MS, were highlighted for the first time in the work of Cecchi and Alfei, 2013 [105]. In this study, the importance of some identified volatile compounds and terpene hydrocarbons, as geographical origin and genotype markers for Italian EVOOs, was also discussed. Melucci et al., 2016 [120], proposed the use of flash GC electronic nose (FGC E-nose) and SPME GC MS, together with MVA, as a method to perform a rapid screening of commercial EVOOs characterized by different declared geographical origin. Specifically, the possible discrimination between 100% Italian and non-100%-Italian (EU) oils was considered. In the work of Kosma et al., 2017 [123], SPME–GC MS, and MANOVA/LDA were used to analyse monovarietal (Koroneiki) olive oils from four Greece regions during two harvesting periods in the phase of full ripeness. The identification and evaluation of volatile compounds, fatty acid composition, total phenolic content, and colour parameters was performed to classify the Koroneiki EVOOs samples according to their geographic origin. The results of this study were also considered useful in establishing trademarks of Greek olive oils. Peršurić et al., 2018 [126] used combined GC-MS, MALDI-TOF MS, and NIR spectroscopic data and chemometrics for the authentication of Croatian EVOOs. The discrimination of the geographical origin was attempted looking at the TAG and FA (fatty acids) analytical profiles among the used analytical techniques, MALDI-TOF MS gave more comprehensive information and was therefore suggested as an indispensable method for qualitative and quantitative EVOO characterisation aimed at geographical origin assessment. In the work of Abbatangelo et al., 2019 [127], an SPME-GC-MS technique combined with a series of metal oxide gas sensors (called S3) was used to identify and discriminate Garda PDO EVOOs from the west and east coasts of Garda Lake in Italy. This approach showed a good potential for geographical origin evaluation and allowed analysis of the EVOOs’ main compounds specifically related to quality and traceability. Quintanilla-Casas et al.’s 2020 [132] study exploited HS-SPME-GC-MS data, combined with chemometric analysis, to examine EVOOs from seven countries (Croatia, Slovenia, Spain, Italy, Greece, Morocco, and Turkey). Samples of the same olive cultivar from different countries were correctly classified according to their provenance, evaluating the presence of sesquiterpene hydrocarbons (SHs) as markers of EVOOs geographical origin. Stilo et al., 2021 [133] used MHS-SPME (M = multiple) followed by GC-MS and flame ionization detection (FID) method to analyse Italian EVOO samples from Sicilia, Toscana, and Garda lake areas. This method allowed the identification of useful markers for geographical origin discrimination guaranteeing data traceability and transferability over the years.

#### 3.1.2. LC-MS

As expected, studies related to more recent LC-MS-based methods for EVOO geographical origin assessment appeared later in the literature with respect to GC-MS. Ouni et al., 2011 [114], exploited the use of rapid-resolution (RR) LC, electrospray-ionization, time-of-flight MS (LC-ESI-TOF MS) method to discriminate olive oil samples from the Oueslati cultivar, grown in different areas from central and southern Tunisia. The results showed significant quantitative differences for many phenolic compounds, according to the considered geographic areas. Taamalli et al., 2012 [118,150], used HPLC-ESI-TOF MS for the classification, according to their specific phenolic composition, of Tunisian olive oils (Chemlali cultivar) from different production areas. The quantitative results and cross-validated data showed significant variability between the analysed oil samples according to their geographic origin. Bakhouche et al., 2013 [119], also used (HPLC) ESI-TOF MS analyses for classification of Arbequina EVOOs from several areas in southern Catalonia (Spain). Again, the results showed quantitative differences in a large number of phenolic compounds correlated with the different geographical origins. In the work of Gil-Solsona et al., 2016 [40], the ultra-HPLC coupled with quadrupolar TOF MS (UHPLC-QTOF MS) was applied for the discrimination of different Spanish EVOOs based on their geographical origin. Once again, specific markers, useful for the possible validation of the MS analytical method for EVOO origin discrimination, were found and proposed. Kalogiuri et al., 2018 [124], applied an optimized and validated LC-ESI-QTOF-MS method associated with an integrated non-target screening workflow, for metabolomic profiling of several monovarietal Greek EVOOs from selected geographical areas. Interestingly, this method set different concentration thresholds for significant cultivar-related markers. The discrimination of EVOOs from nine Spanish PDOs based on UHPLC-ESI MS/MS data was reported by Becerra-Herrera et al., 2018 [106]. Information about EVOO cultivars and geographical origin were correlated by MVA to the phenolic profiles’ analytical data. In the work of Mohamed et al., 2018 [125], a non-targeted approach, based on UHPLC-ESI/QTOF MS data, was used to investigate the sterol and phenolic profiles of Tunisian and Italian EVOOs. The polyphenols and sterols best contributing to the geographic origin discrimination of EVOO samples were also quantified according to their specific chemical subclasses. Statistical models allowed for the identification of the best markers for EVOO discrimination between Tunisian and Italian samples. Ghisoni et al., 2019 [107], applied MVA based on coupled UHPLC-QTOF MS data to discriminate Italian EVOO samples from different geographical origins and cultivars according to their phenolic and sterol fingerprints. Dugo et al., 2020 [131], using HPLC coupled to MS and fluorometric detection, respectively, associated with statistical analyses, characterized and discriminated EVOO samples from Portugal, Spain, and Croatia. Bioactive molecules related to different geographical areas, in particular, phenol and tocopherol, were also identified and correlated with antioxidant assays. Lukic et al., 2020 [98], used LC-ESI-MS/MS, LC-ESI-IT-MS, and ^1^H-NMR to differentiate EVOO samples based on the origin of purchase, such as Italian monocultivar PDO with respect to commercially blended EVOOs. The combined use of these MS-based techniques, together with MVA, successfully indicated various chemical markers useful for the discrimination of the two EVOO classes characterized by different heterogeneity of the geographic and pedoclimatic origin. The results were also supported by parallel isotope ratio MS (IRMS) analyses. Nikou et al., 2020 [37], recently used flow injection analysis, magnetic resonance mass spectrometry (FIA-MRMS) data to obtain the metabolic profiles of monovarietal EVOO samples (Koroneiki) collected from the main Greek producing regions. In parallel, an LC-Orbitrap MS platform was used to both verify the efficiency of the method and increase the security of identification of the proposed markers. With FIA-MRMS, statistically significant compounds and chemical classes were identified as markers of quality and authenticity and associated with specific EVOO characteristics, i.e., geographic region, cultivation practice, and manufacturing procedure. Very recently, Kritokou et al., 2021 [134], found significant differences between Greek EVOOs from five islands originating from the North Aegean Region (Chios, Fournoi, Ikaria, Lesvos, and Samos) using the UHPLC-QTOF-MS method. The biophenol contents were analysed to investigate discriminations between different regions. In addition, Lechhab et al., 2021 [135], detected phenolic compounds, as well as five Vitamin E isomers, using HPLC–PDA-ESI–MS and NP (normal phase) HPLC-FLD. Moroccan EVOOs were discriminated in five zones, indicating the discrimination of olive oil quality in terms of geographical origin as well as influence of the pedoclimatic factors, the crop year of production, and the harvest time.

#### 3.1.3. Both GC and LC-MS

Combined GC-MS and LC-MS methods were recently described for the EVOO geographical origin assessment. Lukic et al., 2019 [128], exploited GC-IT MS and UPLC with diode array detection (DAD), complemented with sensory analysis, for studying the inter-varietal diversity of typical volatile and phenolic profiles of typical Croatian EVOOs from specific geographical locations. Many potential varietal markers were identified by uni- and multi-variate statistical analysis. In the work of Olmo-García et al., 2019 [129], data from two different platforms, LC-ESI-QTOF-MS and GC-APCI-QTOF-MS, combined with chemometric analyses, were used to characterize EVOO samples of six different areas from four Mediterranean countries (Priego de Cordoba and Baena (Spain), Kalamata (Greece), Toscano (Italy), and Ouazzane and Meknes (Morocco)). The contribution of a few thousand molecular characteristics to authenticate the declared origin of commercial EVOOs was evaluated from statistical models. Different compounds were highlighted as possible markers of origin, describing characteristic compositional patterns for each geographical area in the evaluated harvesting year. Crizel et al., 2020 [130], analysed EVOO samples from different cultivars grown in Southern Brazil, from two harvesting years, using GC-MS and UHPLC-QTOF-MS. The obtained data supported the establishment of a local EVOO profile, and the compounds that most contributed to the differentiation could allow the discrimination of samples based on their specific geographical origin, cultivar, and harvesting year.

#### 3.1.4. Others (PTR and MALDI MS)

Specific ionization techniques such as PTR-MS and MALDI were also used for the geographical origin assessment of EVOOs. In the study by Araghipour et al., 2008 [111], the proton-transfer-reaction MS (PTR-MS) was used to classify EVOOs from five different European countries (Italy, Greek, Cyprus, Spain, and France) according to their geographical origins. In this case, the MS data fingerprints for the volatile compounds of samples were used for MVA. Interestingly, Italian EVOO samples could be also further discriminated according to their specific origin, based on smaller regional scale. As shown in the work of Peršurić et al., 2017 [121], the use of a spiral-matrix-assisted laser desorption/ionization (MALDI) TOF MS platform for the assessment of triacylglycerol (TAG) allowed a differentiation of olive oils from different geographical origins. The obtained data were used for statistical surveys aimed at assessing the authenticity of Croatian olive oils. Jergović et al., 2017 [122], used MALDI-TOF MS, coupled with statistical analysis, for the fingerprinting of TAG profiles, useful for detecting adulteration in EVOO samples from different geographical regions. The results showed that this method can distinguish adulterated oils from EVOOs while allowing some discriminations of geographical origin.

### 3.2. Isotope Ratio MS

In recent years, isotope-ratio mass spectrometry (IRMS) applied to the measurement of stable isotopes, usually C, O, and H, has often been used for product origin assessments [151]. A local variability of different isotope ratios can be used for the purposes of PDO/PGI EVOOs’ geographical characterization [138]. This technique measures the ratio between two stable isotopes of an element in a product in relation to agricultural practices, soil, and climatic conditions influencing the specific crop [152]. Interesting classifications of the investigated products could be obtained by quantifying the isotopes’ ratio deviations with respect to reference standards. The IRMS associated with the statistical analysis allows specific studies to be conducted on the assessment of the products’ geographical origin [153]. This normally occurs using specific statistical models able to discriminate the samples, taking advantage of their isotopic parameters influenced by the geographical origin [143]. Therefore, statistical data highlight the influence of climatic factors such as latitude, average temperature, and humidity [154]. In general, the main advantage of the isotopic ratios is its possible use of old or degraded samples since it refers to elemental isotopes (generally C, O, and H), regardless of the compounds these elements are in. A disadvantages of isotopic ratio-based methods is the limited variables number, defining the “digital fingerprint” and their possible non-univocal interpretation. While powerful, IRMS alone is not able to contribute in detail the origin of oil blends from different geographical areas, hence requiring the use of other supporting analytical techniques such as NMR or MS [143]. Besides the already described multi-approach (IRMS, NMR) work of Camin [85] and Lukic [98], the number of scientific papers essentially dealing with IRMS techniques for the EVOO geographical origin assessment (reported in Table 4) is limited. Spangenberg et al., 1998 [136], first used an approach combining GC/MS and compound-specific isotope analysis (CSIA) through GC coupled to a IRMS via a combustion (C) interface, providing further insights into the control of the purity and geographical origin of oils sold as cold-pressed EVOO with certified origin appellation. This study focused on EVOOs from the major oil-producing Mediterranean regions (Spain, Italy, Greece, and France), and results showed that the carbon isotope composition of individual fatty acids was useful for genuine olive oil characterization and may also be a sensitive marker of paleoclimatic changes in the Mediterranean basin. Baum et al., 2010 [137], used GC-C-IRMS, coupled with PCA, LDA, and HCA, to distinguish between Portuguese and Turkish EVOOs by measuring isotope ratio differences in fatty acid methyl esters (FAMEs). These arise, according to the authors, from several regional influences such as different atmospheric carbon dioxide concentrations and water-use efficiency. It was shown that by modelling data related to three different FAMEs (rather than total isotope ratio of the oil), enhanced resolution of the geographic origin discrimination was obtained. Camin et al., 2010 [138], used IRMS and ICP-MS for the characterisation of authentic PDO and PGI Italian EVOOs (from Trentino to Sicily regions). The results showed a promising geographical discrimination of samples based on stable isotope ratios of C, O, and H and mineral composition data. In the multidisciplinary approach used in the study by Chiavaro et al., 2011 [139], IRMS and other conventional techniques were used to evaluate EVOOs from two different Mediterranean areas (Italy and Croatia). The isotopic composition was shown to discriminate among northern, central, and southern Italian regions as well as Croatia and Istria peninsula areas. The observed differentiation was related to climatic conditions typical of the different geographical zones. Faberi et al., 2014 [140], demonstrated the utility of IRMS, associated with MVA, as a tool for origin discrimination of unknown EVOOs samples. In this work, the evaluation of the FAME composition as well as the determination of C stable isotopes ratio, both in bulk oils and in main FAME constituents, were used as a tool for geographical discrimination of Italian PDO/PGI samples. Results showed that ^13^C isotopic values are a robust marker of origin with respect to fatty acid composition. Chiocchini et al., 2016 [141], applied the IRMS technique for authentication and verification of the EVOOs’ geographical origins from four Italian areas (north, south-central Tyrrhenian, central Adriatic, and islands). This study evaluated the most significant large-scale drivers for the isotopic composition of Italian EVOOs that are possibly useful for geographical origin assessment. Portarena et al., 2017 [142], introduced the combined use of IRMS and resonance Raman spectroscopy (RRS) as a promising tool for discriminating EVOOs from production sites that are affected by similar geographic and climatic parameters. In particular, in this study’s EVOO samples from seven Italian coastal regions were analysed and correctly classified, determining their isotopic composition and carotenoid content. Bontempo et al., 2019 [143], used IRMS coupled with GC to distinguish samples originating in EU with respect to non-EU areas. It was shown that, when bulk data were combined with fatty acid isotopic data, the differentiation power of the method clearly improved. Jimenez-Morillo et al., 2020 [144], used an elemental analyser coupled to an IRMS, showing that the assessment of EVOOs isotopic composition provide information not only on the samples geographical origin, but also on the environmental conditions. Interestingly, the stable isotope contents (C, O, H) correlated to the specific geoclimatic conditions of the studied Portuguese geographical area. Very recently, Tarapoulouzi et al., 2021 [145], achieved geographical discrimination among Central Greece and Peloponnese EVOOs, using IRMS measurements. A statistical model able to discriminate olive oils based on geographical origin was obtained with a successful discrimination ability at around 91%.

### 3.3. Elemental Profiling MS

Extended elemental profiles, usually obtained by inductively coupled plasma-mass spectrometry (ICP-MS), were also shown to be useful for food classification, including EVOO geographical origin evaluation [155]. ICP-MS is a well-established technique for elemental profiling (including trace elements) as well as for isotopic determinations. This technique allows rapid analysis of a large range of samples, requires minimal sample preparation, and can be applied to the detection and quantification of a wider range of elements. ICP-MS is also characterised by a good precision for isotope analyses. The increasing availability of multicollector ICP-MS instruments allows a wide range of heavier stable isotope ratio measurements for the authentication of food products [33]. The potential of multielement profiling is proven by the large number of studies on different foodstuffs [155]. On the other hand, focusing specifically on ICP-MS used for EVOO geographical origin assessment, besides the already-mentioned work of Camin [138], there are very few and recently published works (Table 4). Using inductively coupled plasma MS and plasma optical emission spectrometer analysis (ICP-MS/OES), Sayago et al., 2018 [146], explored the potential of multi-element fingerprinting in combination with advanced data mining strategies to assess the geographic origin of EVOOs from several Spanish zones. The MVA results showed that the studied EVOOs exhibited specific element profiles, allowing for differentiation of samples based on their geographical origin. As suggested by Pošćić et al., 2019 [147], ICP-MS was useful in the detection of a large number of elements characterizing EVOO samples originating along the coast of Croatia for application in traceability studies and determination of the geographical origin of the oil. In order to guarantee the control of the geographical origin of the product, Aceto et al., 2019 [148], focused their investigations on microelement determination by ICP-MS analysis. In particular, their study concerned the lanthanide evaluation for production chain tracing of particularly valuable EVOOs from Liguria (Italy) obtained using the Taggiasca cultivar. This method, which is associated with MVA, was considered advantageous and less time-consuming with respect to other techniques used in previous studies for Ligurian EVOOs discrimination [73]. Wali et al., 2021 [149], studied, using ICP-MS and chemometric analysis, the effects of geographic origin and cultivar on oxidative stability and elemental analysis of Tunisian EVOOs. The metal content profiles in EVOO showed significant differences according to the considered regions and cultivars. This study also revealed that the mineral contents in EVOO produced in north of Tunisia (Beja and Zaghouan) were higher than those in the EVOO produced in the south (Sfax).

## 4. Chemometrics

**Figure 4:**
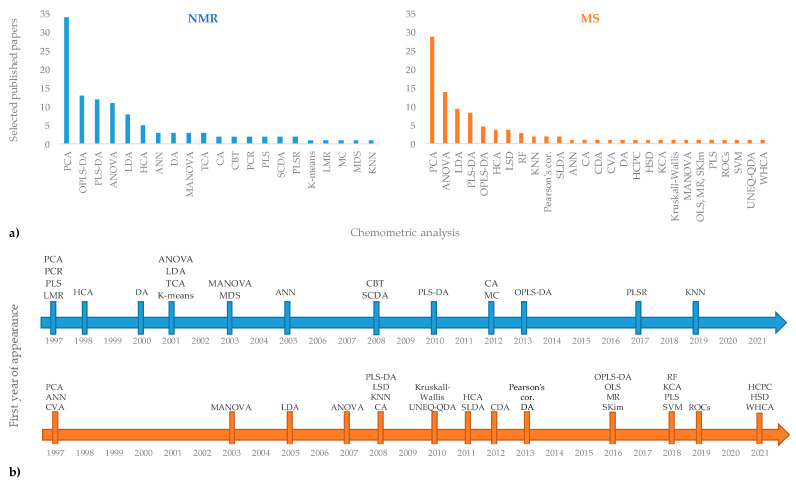
Chemometric analyses associated with NMR and MS techniques in selected published papers reported according to the frequency of their specific use (**a**) and their first appearance in the literature reviewed here (**b**).

The use of different analytical techniques in the geographical origin assessment of EVOOs provide a large number of raw data. Usually, univariate analyses such as analysis of variance (ANOVA and *t*-test) are used for data treatment. However, the need to extract significant information from a huge number of data often requires the use of multivariate methods. Chemometrics can be defined as a science which uses mathematical, statistical, and logical methods to extract information from chemical systems [156]. Among chemometric techniques, unsupervised multivariate analyses (MVA) are commonly used as exploratory methods without any “a priori” knowledge of groups present in the population. Among these, hierarchical clustering analyses (HCA) and Principal Component Analysis (PCA) are commonly used in foodomics. PCA is at the basis of the multivariate analysis [157] and is one of the most common ways to reduce collinearity [158]. A PCA model provides a summary or overview for all samples’ observation in a data table without prior class attribution. Groupings, trends, and outliers can also be found. HCA is a suitable method to obtain an overview of sample clusters providing an easy interpretation of the data [159]. Instead, supervised methods make use of class attribution for samples and are characterized by a powerful predictive ability that is useful for the classification of new data. In these last approaches, linear (such as LDA, PLS-DA, OPLS-DA) and non-linear (e.g., SVM, RF, ANN) methods are profitably used [160,161]. Among these, PLS-DA [162] allows a maximum separation to be obtained between the classes and to obtain information on the variables responsible for the observed separation. The OPLS-DA represents a further evolution of the PLS-DA, separating the portion of the variance useful for predictive purposes from the portion of the non-predictive variance, which is made orthogonal [163]. Unsupervised and supervised multivariate analyses are generally required to manage the complex datasets [162,164,165,166,167]. Specifically, the chemometric applications on NMR and MS techniques for extra-virgin olive oil [160,161] authenticity assessment have been recently described. The chemometric analyses associated with NMR and MS techniques in studies based on geographical origin assessment of EVOOs, discussed in the present review, are reported in Figure 4. Looking at the frequency of their use (Figure 4a), PCA seems to be the most commonly exploited among the chemometric treatments providing a preliminary indication of the role played by the variables used (spectra data attributable to specific buckets) in the discrimination of the samples and in their possible grouping into clusters. Then, PLS-DA and OPLS-DA are commonly used as supervised investigation, in addition to unsupervised analysis, generally for both considered techniques (NMR and MS). Other types of statistical analysis, based on the specific purpose of the considered research, are used less frequently. Considering their first appearance in the literature reviewed here (Figure 4b), PCA dates back to the year 1997, together with PCR, PLS, and LMS in the case of the NMR analytical approach and ANN and CVA for the MS method. Instead, PLS-DA and OPLS-DA are dated later in terms of their first year of appearance (2008/2010 and 2013/2016, respectively), showing that these statistical survey techniques have been used more recently. The last ones used in the studies considered here are KNN for NMR and HCPC, HSD, and WHCA for MS techniques. The most important advantages and limitations of general chemometric methods have been already fully described in the literature [160,161]. A specific description for chemometric treatments used in the papers reviewed in the present work is summarized in Table 6.

## 5. General Remarks

**Figure 5:**
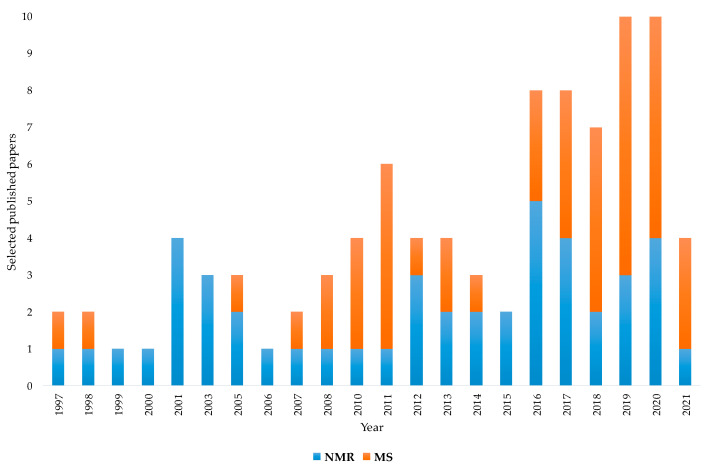
Temporal distribution from 1997 to 2021 (to date) of selected NMR- and MS-based studies for geographical origin assessment of EVOOs. Data from systematic research on https://www.scopus.com/ and https://scholar.google.com/ (accessed on 1 December 2021).

The aim of this review was to present the contribution of NMR and MS spectroscopy, associated with chemometrics, for the assessment of EVOOs’ geographical origin. The examined analytical techniques appear represented in the literature by many papers focused on this specific topic. According to the bibliometric data, the increasing applications of these two techniques over the last couple of decades is characterized by a nearly constant NMR contribution buttressed by a substantial albeit more randomly distributed MS use. Although the first papers on geographical origin assessment appeared in the late 1990s for both NMR and MS, MS applications have become increasingly important in more recent years, reaching and in some cases exceeding the number of publications related to NMR. In particular, as shown in Figure 5, which numerically summarises the previously discussed papers, the temporal distribution from 1997 to 2021 (until this work) highlights how NMR and MS have been increasingly used in recent years. As already discussed, a parallel growing use of sophisticated chemometric methods has been observed in literature. This historical analysis may help to understand the close link between the increasing concern for the assessment of EVOOs’ geographical origin and the available instrumental and statistical tools that are useful for providing reliable solutions.

**Figure 6:**
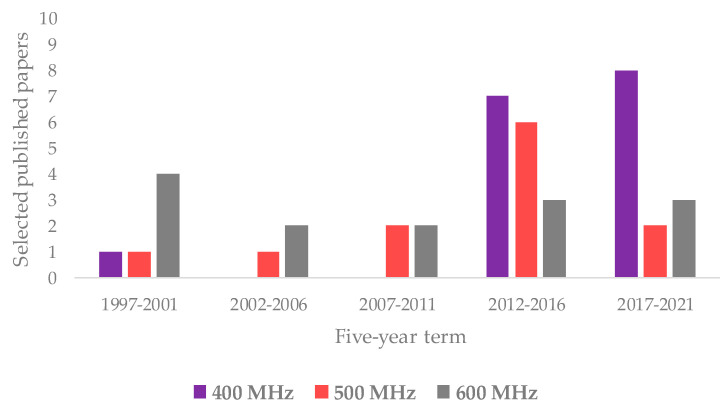
Evolution over five-year period of main ^1^H NMR frequencies (400, 500, and 600 MHz) reported in here selected studies.

Regarding the NMR technique, the works exploiting ^1^H are much more frequent than those related to ^13^C NMR (40 and 12 are listed in this review, respectively); very few also use the ^31^P (2). An overview on the NMR spectroscopies used in the analysis of olive oil has already been reported in the literature [35,168]. As shown by the data reported in this review, ^1^H appears to be the most commonly used nucleus for NMR-based studies on assessment of EVOOs’ geographical origin. Interestingly, the distribution of the used instrument field considerably varied over time. In particular, the ^1^H NMR based on 400 MHz frequency was shown to be the most used in recent years as opposed to the 500 and 600 MHz instruments, as shown in Figure 6. This could be related not only to the improved quality of low field instruments’ performance but also to cost-effective benefits [169]. Interestingly, a 400 MHz instrument dedicated to the food screening task has been specifically produced in recent years [170].

**Figure 7:**
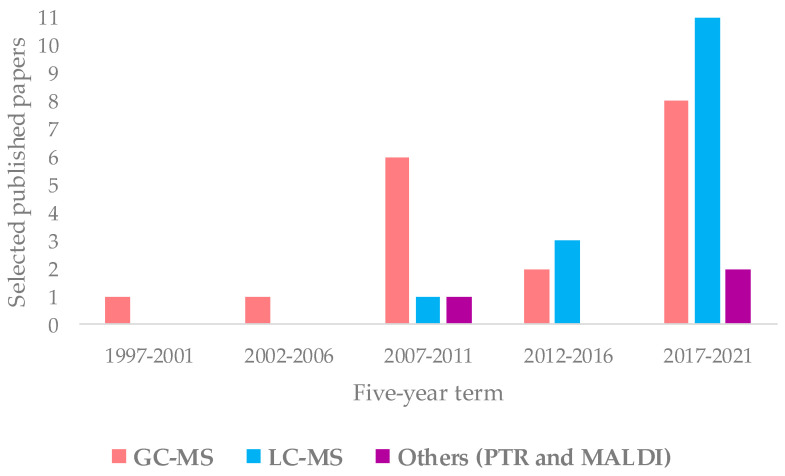
Evolution over five-year period of molecular-fingerprint and chromatography MS techniques reported in here selected studies.

Regarding the MS technique, there are many works concerning the molecular fingerprinting (34) and fewer works on MS-based isotope ratio and elemental profiling analyses (10 and 4, respectively). The characteristics of these different approaches and their comparison are well described in the literature [138,171,172,173]. In geographical origin assessment of EVOOs, the molecular-fingerprint MS studies are essentially based on upstream chromatography approach. The evolution over the time of GC-MS and LC-MS, as well as other ionization techniques such as PTR and MALDI, is shown in Figure 7. The data demonstrate a considerable use of GC (18) and LC-MS (15). In particular, the use of LC-MS considerably increased in recent years, becoming dominant among MS-coupled chromatographic techniques. The advantages in the application of the two most popular methods (GC-MS, LC-MS) are related to their powerful capability of specific metabolites identification [171]. Other kind of MS approaches (such as PTR and MALDI) appeared only in a few studies (3).

**Figure 8:**
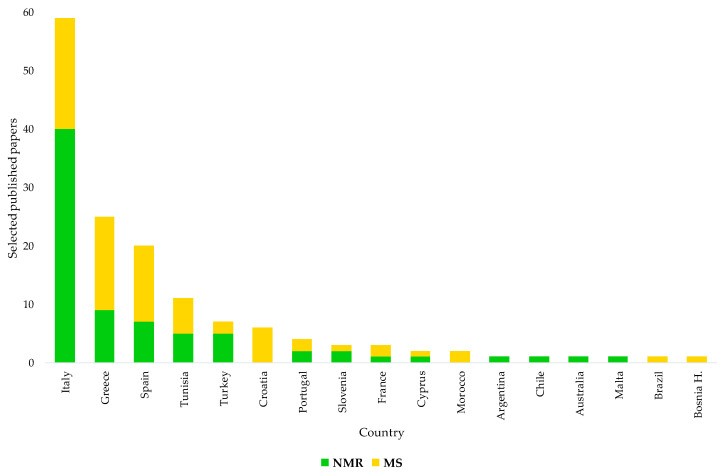
Countries of origins for EVOOs subject to geographical origin assessment in selected NMR and MS-based studies. Data from systematic research on https://www.scopus.com/ and https://scholar.google.com/ (accessed on 1 December 2021).

This review also shows that EVOOs of some specific countries, corresponding to the main worldwide producers essentially located within the Mediterranean area (Spain, Italy, Greece, etc.), have been shown more than others in terms of origin assessment and certification studies (Figure 8). Nevertheless, some specific producers of EVOOs such as Italy have been particularly focused on such studies over the years, possibly because of the consumers’ interest and/or intrinsic economical value. This interest in Italian EVOOs’ origin assessment is also testified by the national concern on the topic. Since 2014, after the EC Regulation 182/2009 had introduced the compulsory labelling of EVOOs with their geographical origin, the Italian government committed itself to developing specific tools for the task. This occurred according to a parliament-approved specific agenda [174] suggesting the evaluation of the opportunity to create a database representing the various productions of EVOOs obtained in the various geographical areas of the country. The idea was to characterize and typify the EVOOs using the methodologies provided by the scientific community. Interestingly, NMR was specifically indicated among the methodologies for the study of the characteristics of mono- and multi-varietal EVOOs, making it possible to guarantee the authenticity of the product and define its peculiarities linked to the territory of origin [174]. Solicited to comply with the commitment in response to a specific question on the problem, the Italian Minister of Agriculture made clear, in 2016, both the lack of and need for a laboratory methodology at European level able to guarantee control of the information on the geographical origin reported on the label [175]. In the same year, the Italian parliament committee against fraud suggested the creation of reference databases for EVOOs’ origin assessment for high-quality Italian EVOO protection and also a possible use of NMR in the task [176]. In 2018, the same issue, namely the need for an analytical method for the determination of product origin, was also highlighted at the 51st meeting of the advisory committee on olive oil and table olives of the International Olive Council (IOC) by a representative of Italian producers [177]. The IOC Executive Secretariat indicated that a method developed by the Italian Ministry of Agriculture had been presented to the group of experts a few years earlier but that the project had not received the support of the Council of Members, which had considered that it was not a priority in terms of the objectives of the Organisation. It was also reported that various national methods were available. The interest in the EVOOs’ geographical origin assessment may have been originally limited to specific countries; with new producing regions in a country and new producing countries, we believe that there is a need for well-established authentication procedures.

## 6. Conclusions

The present review focused on literature work demonstrating that NMR and MS, thanks to their high-throughput data description of the evaluated matrices, are efficient analytical techniques for EVOOs’ authentication and geographical origin assessment. These procedures are usually built on an NMR- and/or MS-based complete metabolic fingerprint of EVOOs from different geographical areas supported by MVA and offer examples of countrywide as well as small-region evaluation, including possible support for PDO and PGI product validation. Published scientific papers for geographic origin assessment of EVOOs represent a wealthy and mature field where a general validation method could be selected and established as a reference procedure for the focused task—an important step overcoming the problem of setting a scientifically sound origin assessment scheme as a key factor for EVOOs trade and consumer awareness. Since there are no official methods for such specific EVOOs authentication, NMR and MS techniques should be considered to establish mandatory legislation and specific labelling rules for characterization of EVOO origin and a certification method recognised worldwide.

## Figures and Tables

**Table 1:** Summary of the most important advantages and shortcomings of NMR and MS techniques [42,43].

Analytical Technique	Advantages	Shortcomings
NMR	High reproducibility. Profitably use for nonselective analysis (fingerprinting). Fast measurement. Minimal sample preparation. Non-destructive. Sample storage for a long time. Inherently quantitative. Correlation between the NMR signal intensity and metabolite concentrations. Suitable for untargeted and targeted analyses.	Intrinsically low sensitivity (improvable with multiple scans, higher magnet field strength, cryo-cooled microprobes, and hyperpolarization methods). Peak overlapping from multiple detected metabolites. Spectral resolution (usually less than 200 metabolites can be unambiguously detected and identified in one measurement).
MS	High sensitivity. Profitable use for selective analysis (in combination with chromatography). Very fast measurement. High number of detected and identified metabolites.	Low reproducibility (compared to NMR spectroscopy). Requirement of a prior sample separation with chromatography. Different ionization methods in sample measurement. Destructive. Sample cannot be recovered. Usually no correlation between the MS line intensity and metabolite concentrations.

**Table 2:** Geographical classification studies of EVOOs by NMR spectroscopy, according to the chronological order of appearance.

Frequency	Nucleus	Geographical Area	Chemometric Treatment	Outcomes *			Year	References
				A	B	C		
75.5 MHz	^13^C	12 Italian Regions	PCA, PLS, PCR, LMR	√	√	√	1997	[58]
600 MHz	^1^H	4 Italian Regions	PCA, HCA	√	-	√	1998	[59]
75.5 MHz	^13^C	3 Italian Regions	PCA, PLS, PCR	√	√	-	1999	[60]
400, 500 MHz	^1^H	Apulia Region (Italy)	PCA, HCA, DA	√	-	√	2000	[61]
75.5 MHz	^13^C	13 Italian Regions PDO	PCA	-	-	√	2001	[62]
600 MHz	^1^H	Tuscany Region (Italy) PDO	HCA, K-means, DA	√	-	-	2001	[63]
600 MHz	^1^H	5 Italian Regions	ANOVA, TCA, LDA	√	-	-	2001	[20]
600, 150.9 MHz	^1^H, ^13^C	Italy and Argentina	TCA, LDA	√	-	√	2001	[64]
150.9 MHz	^13^C	Sicily Region (Italy)	MANOVA, PCA, TCA, MDS, LDA	√	-	√	2003	[65]
125.7 MHz	^13^C	Apulia Region (Italy) PDO	MANOVA, LDA	√	√	√	2003	[66]
125.7 MHz	^13^C	Apulia Region (Italy)	ANOVA, PCA, HCA, DA	√	√	√	2003	[67]
600 MHz	^1^H	Veneto Region (Italy) PDO	ANOVA, PCA	√	-	√	2005	[68]
500 MHz	^1^H	Greece, Italy, Spain, Tunisia, Turkey	LDA, PLS-DA, ANN	√	√	-	2005	[69]
600 MHz	^1^H	Veneto and Lombardia Regions (Italy)	PCA	-	-	√	2006	[70]
600, 62.9 MHz	^1^H, ^13^C	Lazio Region (Italy) PDO	ANOVA, PCA, LDA	√	-	√	2007	[71]
500, 202 MHz	^1^H, ^31^P	2 Greek Regions	SCDA, CBT	√	√	√	2008	[72]
600 MHz	^1^H	Liguria Region (Italy) PDO and other: Italy, Spain, France, Greece, Cyprus, Turkey	PLS-DA	√	√	√	2010	[73]
500 MHz	^1^H	Apulia Region (Italy)	ANOVA, HCA, PCA, LDA	√	-	√	2011	[74]
500, 202 MHz	^1^H, ^31^P	4 Greek Regions	SCDA, CBT	√	-	√	2012	[75]
600 MHz	^1^H	Apulia Region (Italy) PDO, Greece	PCA, CA, MANOVA, MC	√	√	-	2012	[76]
500 MHz	^1^H	Apulia Region (Italy) PDO, Greece, Spain, Tunisia	PCA	-	-	√	2012	[77]
600 MHz	^1^H	Piedmont Region (Italy)	PCA	-	-	√	2012	[78]
500 MHz	^1^H	Apulia Region (Italy)	PCA, OPLS-DA	√	-	√	2013	[79]
400, 500 MHz	^1^H	Turkey, Jordan, Palestine, Libia	ANOVA	-	-	√	2013	[80]
400 MHz	^1^H	Apulia Region (Salento area; Italy)	PCA, OPLS-DA	√	-	√	2014	[81]
400 MHz	^1^H	Apulia and Calabria Regions (Italy) PDO, Greece, Spain	PCA	-	-	√	2014	[82]
400 MHz	^1^H	Apulia Region (Italy)	OPLS-DA	√	-	√	2015	[83]
700 MHz	^1^H, ^13^C	Sicily Region (Italy) PDO	PCA	-	-	√	2015	[84]
600 MHz	^1^H	15 Italian Regions PDO and Tunisia	PCA	-	-	√	2016	[85]
400 MHz	^1^H	Apulia Region (Italy) PDO	PCA, PLS-DA, OPLS-DA	√	√	√	2016	[86]
400 MHz	^1^H	Apulia & Calabria Regions (Italy)	PCA, PLS-DA, OPLS-DA	√	√	√	2016	[87]
400 MHz	^1^H	Apulia Region (Italy)	PCA, PLS-DA, OPLS-DA	√	-	√	2016	[50]
400, 500 MHz	^1^H	Apulia Region (Italy)	PCA, PLS-DA, OPLS-DA	√	-	√	2016	[88]
400 MHz	^1^H	Apulia Region (Italy)	PCA, ANN	√	√	√	2017	[89]
500 MHz	^1^H	Italy (Tuscany, Sicily and Apulia Regions), EU (Spain and Portugal) and non-EU (Tunisia, Turkey, Chile and Australia)	PCA, OPLS-DA	√	-	√	2017	[46]
600 MHz	^1^H	Sardinia Region (Italy)	PCA, OPLS-DA	√	√	√	2017	[90]
400 MHz	^1^H	Tunisia and Italy	PCA, PLS-DA, OPLS-DA, PLSR	√	√	√	2017	[91]
400 MHz	^1^H	Tuscany Region (Italy) PGI	PCA, OPLS-DA	√	√	√	2018	[49]
600 MHz	^1^H	Turkey and Slovenia	ANOVA, PCA, PLS-DA	√	√	√	2018	[92]
600 MHz	^1^H	Italy	LDA	√	√	√	2019	[22]
400 MHz	^1^H	Italy, Greece, Spain	PCA, CA, KNN	√	√	-	2019	[93]
600 MHz	^1^H	Turkey	ANOVA, PLS-DA	√	√	√	2019	[94]
500 MHz	^13^C	8 Italian Regions	ANOVA, PCA	-	-	-	2019	[95]
400 MHz	^1^H	Italy	PCA, PLS-DA, OPLS-DA	√	√	√	2020	[23]
400 MHz	^1^H, ^13^C	Tuscany Region (Italy)	ANOVA, PCA	-	-	√	2020	[96]
500 MHz	^1^H, ^13^C	Malta	PCA, PLS-DA, ANN	√	-	√	2020	[97]
400 MHz	^1^H	Italy (also PDO)	ANOVA, PCA, PLS-DA	√	-	√	2020	[98]
400 MHz	^1^H	International Blends (Italy, Tunisia, Portugal, Spain, Greece)	PCA, PLSR, OPLS-DA	√	√	√	2021	[99]

* Summarized outcomes for the listed NMR studies: A Classification model realization; B Prediction test execution; C Molecular markers identification.

**Table 3:** Key molecular markers identified in NMR studies of Table 2.

Molecular Markers *	References	Molecular Markers *	References
Aldehydes	[64,70,71,81,82,83,89,92,94]	n-Alkanals	[59]
Carotenoids	[81,96]	Oleacein and Oleocanthal	[23,99]
cis-Vaccenis acid	[64,65]	Oleic Acid	[23,46,49,50,58,62,64,65,66,67,71,72,74,77,79,80,84,86,87,88,91,98,99]
Coumaric acid	[75]	Peroxides	[23,99]
Cycloartenol	[70,92]	Phenolic Compounds	[72,75,81,82,83,89,92]
Eicosenoic acid	[64,65]	Pigments	[96]
Elenolic acid	[99]	Pinoresinol	[72,75]
Flavonoids (including Apigenin and Luteolin)	[72,75]	Satured Fatty Acids	[22,23,46,49,50,58,62,64,65,66,67,71,73,77,79,80,83,84,85,86,87,88,90,98,99]
Formaldehyde	[64]	Secoiridoids	[61,96]
Hexanal	[22,64,68,70,73,90]	Squalene	[22,23,64,68,71,73,78,80,84,85,90,92,96,97]
Homovanillic acid	[75]	Sterols (including β Sitosterol)	[22,59,64,67,71,72,73,85,92,97]
Hydroxityrosol	[23,72,75,82,99]	Syringaresinol	[72,75]
Linoleic Acid	[22,23,46,49,50,58,62,64,65,66,67,72,73,74,75,77,78,79,80,81,83,84,85,86,87,91,98,99]	Terpenes	[22,64,68,70,71,73,74,78,92,94,97,98]
Linolenic Acid	[22,23,46,49,50,64,67,68,73,75,77,78,79,80,81,83,84,86,87,90,91,98,99]	Trans-2-Alkenals	[59]
Methyl cyclohexanol	[22]	Trans-2-Hexenal	[22,64,68,70,73,78,90]
Mono/Di/Tri-acylglycerols	[22,67,68,72,73,85,89,90,92,98]	Tyrosol	[23,72,75,82,99]
MUFA	[49,58]	Volatile Compounds	[59,71,90]

* as defined in the specific referenced papers.

**Table 4:** Geographical classification studies of EVOOs by MS, according to the chronological order of appearance.

Combined Approach	Geographical Area	Chemometric Treatment	Outcomes *			Year	References
			A	B	C		
MS MOLECULAR FINGERPRINT							
Pyrolysis MS	Italy	ANN, PCA, CVA	√	√	-	1997	[108]
HS-MS	Italy, Greece, Spain, Tunisia, commercial EVOOs	PCA, LDA	√	√	√	2005	[109]
GC-CI-ITD MS	Calabria Region (Italy) and Tunisia	LDA, ANOVA	√	-	√	2007	[110]
PTR-MS	Italy, Greece, Cyprus, Spain, France PDO	ANOVA, LSD, PLS-DA	√	-	√	2008	[111]
HS-MS	Spain and Italy PDO	KNN, CA, PCA	√	√	-	2008	[112]
HS-MS, UV–vis, NIR	Liguria Region (Italy)	PCA, UNEQ-QDA	√	-	-	2010	[113]
RRLC-ESI-TOF-MS	Central and Southern Tunisia	ANOVA, PCA, HCA	-	-	√	2011	[114]
HS-SPME-GC/MS	Western Greece	ANOVA, LDA, PCA	√	-	√	2011	[115]
HS-SPME-GC/MS	Spain PDO	LDA, PCA, SLDA	√	√	√	2011	[116]
SPME-GC/MS	Crete and Tunisia	ANOVA, PCA	-	-	√	2011	[117]
HPLC-ESI-TOF-MS	Tunisia	ANOVA, CDA	√	√	√	2012	[118]
HPLC-ESI-TOF-MS	Southern Catalonia (Spain)	DA	√	√	√	2013	[119]
HS-SPME–GC–MS	Italy	Linear regressions, Pearson’s correlations (r), standard deviations.	-	-	√	2013	[105]
FGC E-nose, SPME/GC-MS	Italy PGI and PDO and non-Italy	PCA, HCA, LDA, PLS-DA	√	√	√	2016	[120]
UHPLC-QTOF-MS	Spain	PLS-DA, OPLS-DA	√	√	√	2016	[40]
MALDI-TOF MS	Croatia	PCA	-	-	√	2017	[121]
MALDI-TOF MS	Northwest Istria, Dalmatia, Italy and Bosnia and Herzegovina	PCA	-	-	√	2017	[122]
SPME/GC-MS	Greece	MANOVA, LDA	√	-	√	2017	[123]
LC-ESI-QTOF-MS	Greece	PCA, RF	√	-	√	2018	[124]
UHPLC-ESI-MS/MS	Spain PDO	PCA, LDA	√	-	√	2018	[106]
UHPLC-ESI-MS/QTOF MS	Tunisia and Italy	OPLS-DA, KCA	√	-	√	2018	[125]
GC-MS, MALDI-TOF/MS, NIR	Croatia	PCA, PLS-DA, PLS	√	√	√	2018	[126]
SPME-GC-MS	Garda (Italy) PDO	PCA, KNN	√	-	√	2019	[127]
UHPLC-QTOF-MS	Italy	OPLS-DA, HCA	√	√	√	2019	[107]
GC-IT-MS and UPLC-DAD	Croatia	ANOVA, LSD, SLDA, PLS-DA	√	-	√	2019	[128]
LC-ESI-QTOF-MS and GC-APCI-QTOF-MS	6 Mediterranean GIs PDO (from Spain, Greece, Italy and Morocco)	PCA, PLS-DA	√	√	√	2019	[129]
GC-MS, UHPLC-QTOF MS	Southern Brazil	PCA, ANOVA, LSD test	-	-	√	2020	[130]
HPLC-PDA/MS, HPLC-FLD	Italy, Portugal, Spain and Croatia	PCA, LDA	√	-	√	2020	[131]
LC-ESI-MS/MS, LC-ESI-IT-MS, IRMS, ^1^H NMR	Italy PDO and commercial blends	ANOVA, LSD, PCA, PLS-DA	√	-	√	2020	[98]
FIA-MRMS, UPLC-HRMS, HRMS/MS	Greece	PCA, OPLS-DA	√	-	√	2020	[37]
HS-SPME-GC-MS	Croatia, Slovenia, Spain, Italy, Greece, Morocco, Turkey	ANOVA, PCA, PLS-DA	√	-	√	2020	[132]
MHS-SPME, GC-MS, GC-FID	Sicily, Tuscany, and Garda lake Regions (Italy)	PLS-DA	√	-	√	2021	[133]
UHPLC-QTOF-MS	Greece (North Aegean Region)	ANOVA	-	-	√	2021	[134]
HPLC–PDA-ESI–MS and NP-HPLC-FLD	Morocco	PCA, HCPC, Pearson’s correlations, ANOVA, Tukey test (HSD)	√	-	√	2021	[135]
ISOTOPE RATIO IRMS							
IRMS, GC-MS	Spain, Italy, Greece, France	PCA	-	-	-	1998	[136]
GC-C-IRMS	Portugal and Turkey	PCA, LDA, ANOVA, HCA	√	-	-	2010	[137]
IRMS, ICP-MS	Italy PDO and PGI	Kruskall–Wallis and multiple bilateral comparison	-	-	-	2010	[138]
IRMS, HPLC-APCI-MS	Italy and Croatia	ANOVA, LDA	√	-	-	2011	[139]
EA/IRMS, GC/FID	Italy PDO/PGI	PCA, PLS-DA	√	-	-	2014	[140]
IRMS	9 Italian Regions	Regression-geostatics combined approach (OLS, MR, SKlm)	-	√	-	2016	[141]
IRMS and RRS	Italian coasts	PCA, LDA	√	√	-	2017	[142]
GC-C/Py-IRMS	EU and non-EU	PCA, ROCs, RF	√	-	-	2019	[143]
IRMS	Portugal	PCA, LMR	-	-	-	2020	[144]
IRMS	Central Greece and Peloponnese	OPLS-DA	√	√	-	2021	[145]
ELEMENTAL PROFILE ICP-MS							
ICP-MS/OES	Spain	PCA, LDA, PLS-DA, SVM, RF	√	-	-	2018	[146]
ICP-MS	Croatia	ANOVA	-	-	-	2019	[147]
ICP-MS	Liguria Region (Italy)	PCA, LDA	√	-	-	2019	[148]
ICP-MS	Tunisia	PCA, WHCA	-	-	-	2021	[149]

* Summarized outcomes for the listed MS studies: A Classification model realization; B Prediction test execution; C Molecular markers identification.

**Table 5:** Key molecular markers identified in MS studies of Table 4.

Molecular Markers *	References	Molecular Markers *	References
Alcohols	[106,114,117,118,119,128,129]	Ketones	[127,128]
Aldehydes	[127,128]	Lignans (including pinoresinol and syringaresinol)	[37,106,114,118,119,129,134]
Benzenoids	[128]	Ligstroside Aglycone	[114,118,119,129,130,134]
Carbonyl Compounds	[117]	Oleacein	[134]
Carboxylic Acids	[127]	Oleocanthal	[124,134]
Chlorophylls	[130]	Organic acid	[40]
Cholesterol Derivatives	[107,125]	Other Secoiridoids	[37,106,114,118,119,129,130,134,135]
Diglycerides	[37]	Tyrosol	[106,118,119,134]
Elenolic Acid	[114,118,119,129]	Vanillic Acid	[124]
Esters	[117,127,128]	Terpenes (including sesquiterpene)	[120,127,128,132]
Fatty Acids	[37,98]	Vitamin D3 derivates	[40]
Flavonoids (including Apigenin and Luteolin)	[37,107,114,118,119,124,125,129,134,135]	Vitamin E Isomers and derivates	[130,131,135]
Furanoids	[128]	Phenolic Compounds	[106,107,114,118,119,130,131,134,135]
Hydrocarbons	[117,127,128]	Triterpenoids	[37,98]
Hydroxybenzoic Acids	[125]	Triacylglycerols	[37,40,98,121,122,123,126]
Hydroxycinnamics	[107]	Oleuropein and derivatives	[114,118,119,129,130,134]
Hydroxytyrosol	[106,118,119,129,130,134]	Volatile Compounds (including limonene, pentadiene, hexane)	[105,109,110,111,115,116,117,120,123,124,133]

* as defined in the specific referenced papers.

**Table 6:** Summary of the most important advantages and shortcomings of chemometric methods [160,161] related to the works considered in the present review.

Chemometrics	Methods	Advantages	Shortcomings
Unsupervised	PCA	Quick evaluation and data overview.	No class information. The non-linear combination of the variables is not taken into consideration. Requirement of a scaling method. Risk of misleading (principal components explained lower variance).
	HCA	Quick sample cluster overview. Easy interpretation of results.	No class information. Sensitivity to outliers. No easy derivation of variable importance. Time-consuming.
Supervised	LDA	Easy, simple, and fast data overview. Suitable for linear and low-dimensional data.	Lost of sensitivity in multi-classification task. Not suitable for higher-dimensional data. Non-linear information between the classes and the variables is not taken into consideration.
	PLS-DA	Quick derivation of important variables in peaks list. Suitable for linear data. Usefulness to handle the collinearity among the variables.	Non-linear information of the peaks list is not taken into consideration. Requirement of a scaling method.
	OPLS-DA	Easy interpretation of the models. Usefulness for biomarker discovery.	Non-linear information between the peaks list and classes of the samples are not taken into consideration. Requirement of a scaling method.
	RF	Easy interpretation of results. Usefulness for multi-classification task. No requirement of a scaling method.	Vulnerable decision trees. Requirement of a large sample size.
	ANN	Easy interpretation of results.	Time consuming. Complex training and validation procedure. Requirement of a scaling method. Difficult interpretation of models.

## Data Availability

As this manuscript is a review article, data was from compilation of previously related studies, coming from various authors.

## Abbreviations

ANN, artificial neural networks; ANOVA, analysis of variance; AOCS, American Oil Chemists Society; AOOPA, American Olive Oil Producers Association; APCI, atmospheric pressure chemical ionization; C, combustion; CA, canonical analysis; CBT, classification binary trees; CSIA, compound-specific isotope analysis; 2D, two dimensional; D, discriminant analysis; EC, European Commission; EEC, European Economic Community; ES, electrospray; ESI, electrospray ionization; EU, European Union; EVOO, extra virgin olive oil; FA, fatty acids; FAMEs, fatty acids methyl esters; FGC, E-nose flash gas chromatography electronic nose; FIA, flow injection analysis; FID, flame ionization detection; FT, Fourier transform; GC, gas chromatography; HCA, hierarchical cluster analysis; HCPC, hierarchical clustering on principal components; HPLC, high-performance liquid chromatography; HR-MAS, high-resolution magic angle spinning; HS, headspace; HSD, Tukey’s honestly significant difference test; ICP, inductively coupled plasma; IOC, International Olive Council; IRMS, isotope ratio mass spectroscopy; IT, ion trap; JAS, Japan Industrial Standards; K-means, k-means clustering; KNN, *K*-nearest neighbour; LC, liquid chromatography; LDA, linear DA; LMR, linear multiple regression; LSD, least-significant difference test; MALDI, matrix-assisted laser desorption/ionization; MANOVA, multivariate analysis of variance; MC, Monte Carlo validation approach; MDS, multidimensional scaling; MHS, multiple headspace; MR, multiple regression; MRMS, magnetic resonance mass spectrometry; MS, mass spectrometry; MVA, multivariate statistical analysis; NAOOA, North American Olive Oil Association; NIR, near-infrared spectroscopy; NMR, nuclear magnetic resonance; OES, optical emission spectrometer; OLS, ordinary least squares; OPLS-DA, orthogonal projections to latent structures discriminant analysis; PCA, principal component analysis; PCR, principal component regression; PDO, denomination of protected origin; PGI, Protected Geographical Indication; PLS, partial least squares; PLS-DA, partial least squares-discriminant analysis; PTR, proton transfer reaction; QTOF, quadrupole time of flight; RF, random forest; ROCs, receiver operating characteristic curves; RR, rapid resolution; RRS, resonance Raman spectroscopy; SCDA, stepwise canonical DA; SIMCA, soft independent modelling and class analogy; SKlm, simple kriging with local means; SLDA, stepwise linear discriminant analysis; SPME, solid-phase micro extraction; SVM, support vector machine; TAG, triacylglycerol; TCA, tree cluster analysis; TOF, time of flight; UHPLC, ultra-high-performance liquid chromatography; UNEQ-QDA, unequal-quadratic discriminant analysis; US, United States; UV, ultraviolet; WHCA, Ward’s hierarchical agglomerative clustering analysis.
